# Supplementary material for: Clinical outcome of neurological patients with COVID-19: the impact of healthcare organization improvement between waves
Source: Neurol Sci. 2022 Feb 17;43(5):2923–7. doi: 10.1007/s10072-022-05946-8 (PMC8852998; doi:10.1007/s10072-022-05946-8)
Supplement: Supplementary file 1 — Supplementary file1 (DOCX 382 KB) [file 10072_2022_5946_MOESM1_ESM.docx]

**Supplementary figure 1** Cox Regression model evaluating the impact of hospitalization period (i.e first vs second pandemic wave, Panel A) and Steroid Treatment (Panel B). Data are corrected for the effect of age, sex, premorbid comorbidity index, COVID-19 disease severity.


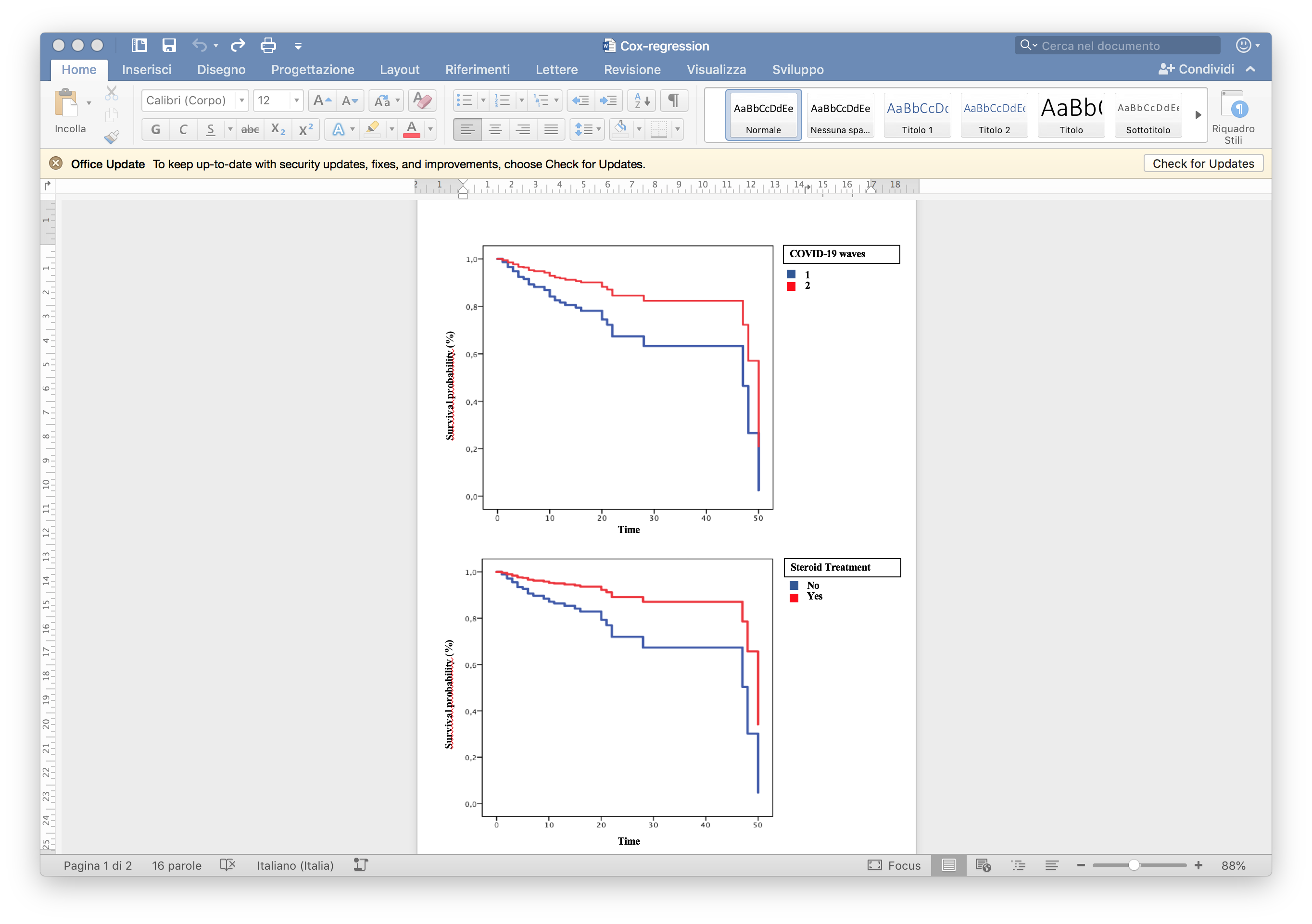


**Supplementrary Table 1** Neurological diagnosis distribution during the first and second pandemic waves. Abbreviations: GBS, Guillain-Barrè syndrome; ICH, Intracerebral haemorrhage, SAH, Subarachnoid haemorrhage; TIA: transient ischemic attack.

|  | **NeuroCOVID** | **NeuroCOVID** | **NeuroCOVID** | ***p Value** |
| --- | --- | --- | --- | --- |
|  | **Total (n=223)** | **1° wave** | **2° wave** |  |
|  |  | **(n=112)** | **(n=111)** |  |
| **Admitting diagnosis** |  |  |  |  |
| Stroke | 79 (35.4%) | 54 (48.2%) | 25 (22.5%) | **<0.001** |
| ICH | 14 (6.3%) | 11 (9.9%) | 3 (2.7%) | 0.050 |
| SAH | 7 (3.1%) | 2 (1.8%) | 5 (4.5%) | 0.280 |
| TIA | 15 (6.8%) | 8 (7.2%) | 7 (6.3%) | 0.789 |
| Seizures | 20 (9.0%) | 9 (8.0%) | 12 (10.8%) | 0.501 |
| encephalitis | 13 (5.8%) | 4 (3.6%) | 9 (8.1%) | 0.166 |
| Encephalopathy | 14 (6.3%) | 12 (10.7%) | 20 (18.0%) | 0.050 |
| Tumor | 8 (3.6%) | 2 (1.8%) | 6 (5.4%) | 0.171 |
| Headache | 9 (4.0%) | 2 (1.8%) | 7 (6.3%) | 0.101 |
| Dizziness | 3 (1.3%) | 0 | 3 (2.7%) | 0.122 |
| GBS | 9 (4.0%) | 5 (4.5%) | 4 (3.6%) | 0.744 |
| Others | 13 (5.8%) | 3 (2.7%) | 10 (9.0%) | 0.050 |
